# Comment on “*Helicobacter pylori* Outer Membrane Protein 18 (Hp1125) Is Involved in Persistent Colonization by Evading Interferon-***γ*** Signaling”

**DOI:** 10.1155/2015/354519

**Published:** 2015-04-06

**Authors:** Amin Talebi Bezmin Abadi, Enzo Ierardi

**Affiliations:** ^1^Department of Bacteriology, Faculty of Medical Sciences, Tarbiat Modares University, P.O. Box 14115-111, Tehran, Iran; ^2^Division of Gastroenterology, Department of Emergency and Organ Transplantation, Piazza Giulio Cesare, 70124 Bari, Italy

We read with interest the paper by Shan et al. [1] in a recent issue. It is an interesting paper concluding that* Helicobacter pylori* (*H. pylori*) omp18 is indirectly affecting long term bacterial colonization by successfully influencing IFN-*γ*-mediated immune response. Nevertheless, we found that some statements could not support the final conclusion.* H. pylori* infects the gastric mucosal layer of half of the human population worldwide and causes various digestive disorders such as chronic gastritis, gastric ulcer, duodenal ulcer, and gastric cancer [2]. To date, it has been established that such complex mechanism of bacterial interaction with human host can shape the successful and persistent colonization of* H. pylori* [3, 4]. Undoubtedly, understanding the mechanisms of immune evasion could provide new options for better management of infection. To our knowledge, the host immune response to the infection is ineffective; accordingly, the bacterium persists and remains for decades. In brief, Shan et al. [1] reported the* oipA* as a critical factor affecting bacterial colonization. However, we know that, in chronic process of colonization adopted by* H. pylori*, the connection of a unique factor to the drive of the final pattern of this phenomenon could be too speculative. Despite the interesting report of Shan et al. [1], we may hypothesize more factors involved in* H. pylori* colonization. Surprisingly,* H. pylori* colonization is not comparable with that of other pathogens [5]. Indeed, different mechanisms are contributing to this mysterious and long term biologic function. Conclusively, more studies are necessary to draw a direct and final conclusion on “the mystery” of* H. pylori* colonization.
